# Menstrual hygiene management in rural schools of Zambia: a descriptive study of knowledge, experiences and challenges faced by schoolgirls

**DOI:** 10.1186/s12889-018-6360-2

**Published:** 2019-01-05

**Authors:** Joyce Chinyama, Jenala Chipungu, Cheryl Rudd, Mercy Mwale, Lavuun Verstraete, Charity Sikamo, Wilbroad Mutale, Roma Chilengi, Anjali Sharma

**Affiliations:** 1 Centre for Infectious Disease Research in Zambia, P.O Box 34681, Lusaka, Zambia; 2 United Nations Children’s Fund, P.O Box 33610, Lusaka, Zambia; 3 Department of Public Health, Section of Health promotion, School of Medicine, University of Zambia, P.O Box 50110, Lusaka, Zambia

**Keywords:** Menstrual hygiene management, Rural schools, Adolescent girls, Zambia

## Abstract

**Introduction:**

While in school, girls require an environment that is supportive of menstrual hygiene management (MHM) in order to ensure regular school attendance and participation. Little is known about schoolgirls access to and practice of MHM in rural Zambia. This study explores girls’ experiences of MHM in rural schools of Zambia from the perspectives of schoolgirls, schoolboys and community and school-based adults key to MHM for schoolgirls.

**Methods:**

In July and August 2015, we conducted this qualitative exploratory study in six rural schools of Mumbwa and Rufunsa districts of Zambia. Twelve in-depth interviews (IDIs) and six focus group discussions (FGDs) were conducted among girls ages 14–18 who had begun menstruating. Two FGDs with boys ages 14–18 and 25 key informant interviews were also conducted with teachers, female guardians and traditional leaders to provide the context within which schoolgirls practice MHM.

**Results:**

Most girls reported learning about menstruation only at menarche and did not know the physiological basis of menstruation. They reported MHM-related challenges, including: use of non-absorbent and uncomfortable menstrual cloth and inadequate provision of sanitary materials, water, hygiene and sanitation facilities (WASH) in schools. In particular, toilets did not have soap and water or doors and locks for privacy and had a bad odor. Girls’ school attendance and participation in physical activities was compromised when menstruating due to fear of teasing (especially by boys) and embarrassment from menstrual leakage. Boys said they could tell when girls were menstruating by the smell and their behaviour, for instance, moving less and isolating themselves from their peers. Girls complained of friction burns on their inner thighs during their long journey to school due to chaffing of wet non-absorbent material used to make menstrual cloth. Girls preferred to dispose used menstrual materials in pit latrines and not waste bins for fear that they could be retrieved for witchcraft against them. Though traditional leaders and female guardians played a pivotal role in teaching girls MHM, they have not resolved challenges to MHM among schoolgirls.

**Conclusion:**

When menstruating, schoolgirls in rural Zambia would rather stay home than be uncomfortable, inactive and embarrassed due to inadequate MHM facilities at school. A friendly and supportive MHM environment that provides education, absorbent sanitary materials and adequate WASH facilities is essential to providing equal opportunity for all girls.

**Electronic supplementary material:**

The online version of this article (10.1186/s12889-018-6360-2) contains supplementary material, which is available to authorized users.

## Background

Water, sanitation and hygiene (WASH) interventions aimed at keeping girls in school in sub-Saharan Africa through provision of sanitary materials, water, soap and privacy show mixed impact on school absenteeism [1, 2]. For instance, a study in Kenya found that cleanliness of school latrines reduced the odds of absenteeism [3] and others suggest that one in ten school-aged girls in low and middle income countries fail to attend school during menstruation or drop out of school at puberty due to the absence of menstrual hygiene management (MHM) facilities [4–7]. Quantitative studies in Ghana and Malawi found moderate to non-significant improvements to school attendance associated with MHM interventions [8, 9]. Nonetheless, WASH for MHM should be considered as a basic right to ensure girls’ comfort, self-confidence and school attendance to reduce gender disparities in education, health and socio-political and economic participation [10, 11].

Menstrual hygiene management requires availability of and access to clean and absorbent menstrual material, privacy, water and soap, and disposal facilities for used menstrual materials [12]. However, most schools in developing countries, especially in rural areas, have inadequate facilities including water supply [13] for girls to wash hands, external genitalia and soiled clothes. Nor do they have provision for privacy, soap, sanitary pads and disposal of soiled sanitary pads [14]. Girls’ participation and psychological well-being while in class is affected when they do not have access to sanitary pads or adequate alternatives because they fear staining their clothes and subsequently being teased and humiliated by their classmates [4, 7]. It is not surprising then, that attendant hormonal disruptions notwithstanding, girls’ school performance has been noted to decline after they attain menarche in Africa [7].

Education of girls directly impacts national health and national development as well as economic and social progress [15, 16]. Educated women tend to have fewer children, lead healthier lifestyles and raise healthier families by making more informed choices [10]. Being more likely to practice and seek appropriate preventive and medical services such as personal hygiene, nutrition and immunization, they help reduce infant morbidity and mortality in the nation [17, 18]. This in turn leads to lowered fertility rates and higher market productivity thereby improving the national economy [10]. In Zambia, however, this potential is cut short with girls dropping out or not attending school, which is reflected in the low female literacy levels of approximately 58%. About 44% of girls are reported to drop out of school before completing their secondary education [19]. One reason for this interruption could be inadequate provision for MHM that does not allow all girls to attend school with dignity and comfort during their menstrual period.

Little attention has been given to understanding the ways in which MHM contributes to school absenteeism and other gender disparities in Zambia. One notable initiative that has been implemented in rural schools of Zambia is the ‘School Led Total Sanitation’ (SLTS) programme. SLTS programmes promote sanitation and hygiene behaviours and provide improvements to the sanitation infrastructure including MHM friendly facilities. Schools with such programmes will most likely have ventilated improved toilets with a covered pit and a designated handwashing facility with a cleaning agent (soap/ash) [20]. Understanding the differences between MHM practices across schools with and without SLTS can provide important insights on the effectiveness of SLTS for MHM. This study was conducted to understand girls’ experiences of managing their menstruation and to further explore how the menstrual hygiene environment within schools may affect their attendance in school.

## Methods

This exploratory qualitative study draws on 12 in-depth interviews (IDIs) and 6 focus group discussions (FGDs) conducted among 14–18 years old girls who had already attained menarche and two FGDs with boys of the same age group. Seven key informant interviews (KIIs) were also conducted with teachers serving as School Health Nutrition (SHN) coordinators who are focal persons for health-related issues including menstruation; 7 with female guardians of girls aged between 14 and 18 years; and 11 with traditional leaders in the communities surrounding the study schools. The interviews and FGDs explored the participants’ perspectives on the experiences of girls in managing menstruation at school. Table 1 below summarises population characteristics and sample size per method.

**Table 1:** Population characteristics, and sampling size by method

Method	Participant Type	Gender	Age	# procedures	# participants
FGD	Pupils	Female	14–18 years	6	48
		Male	14–18 years	2	16
IDI	Pupils	Female	14–18 years	12	12
KII	Teachers	Male and female	> 18 years	7	7
	Guardians	Female	> 18 years	7	7
	Traditional leaders	Male and female	> 18 years	11	11

### Study area

The study was conducted in six schools from two rural districts, Mumbwa located 150 km due west and Rufunsa, located 150 km due east of Lusaka city as selected by the Ministry of Education.

Mumbwa district is located in Central Province of Zambia with an estimated population of 218,328. The population depends on agriculture including a cotton ginnery and maize growing for subsistence. The district is divided into villages headed by traditional chiefs and headmen. There is a total of 148 schools including primary and secondary schools in the entire district. On the other hand, Rufunsa District is located in Eastern Province of Zambia and has a population of 49,337. Like Mumbwa District, Rufunsa is rural, dependent on subsistence farming with villages headed by traditional chiefs and headmen. The district has a total 25 primary and secondary schools. The majority of the population in Rufunsa have no access to modern sanitation.

### Sampling

The six schools in the study were purposively sampled in consultation with the District Education Board Secretary (DEBS) staff to include primary and secondary schools located at varying distances from the DEBS and to include those with and without SLTS interventions to understand their impact on girls’ ability to meet their MHM needs. Schools with long distances to DEBS had poor access to roads, goods and services compared to those located closer to the DEBS.

Meetings were conducted with the Parents’-Teachers’ Association (PTA) for each selected school to introduce the study. Thereafter, female teachers helped identify eligible female pupils who had begun their menses and male pupils who were between 14 and 18 years. Female guardians of the girls identified to participate in FGDs and IDIs were introduced to the study along with the girls. Guardians’ individual consent and adolescents’ individual assent were collected in confidential settings. School principals identified the traditional leaders from their school catchment area and the SHN coordinators at their schools.

### Data collection

Female research assistants with backgrounds in various social science disciplines were trained in human subjects’ protection and research methods by the regulatory and study teams at the Centre for Infectious Disease Research in Zambia. The training included didactic training and role-plays on data collection methods, review of data collection tools and the consenting process. Data collection was conducted in July and August 2015 using field guides for IDIs, KIIs and FGDs (Additional files 1, 2, 3, 4, 5 and 6). A structured observation form was used to assess WASH characteristics around the school. The field guides used in the study were adapted from the WASH in Schools Empowers Girls’ Education-Tools for MHM Booklet [21]. All guides except the structured observation form were translated into Nyanja, the local language spoken in the districts. All interviews and FGDs were recorded using voice recorders with participants’ consent and took between 40 to 120 min with KIIs taking 40–60 min, IDIs 60–90 min and FGDs between 90 and 120 min. The recorded data were transcribed in English by the research assistants who were conversant in Nyanja and English. The Zambian analysts, also fluent in both languages, checked transcriptions against audio recordings rectifying any errors before commencing analysis.

IDIs are a powerful technique in bringing out personal experiences and have been used in qualitative exploration on menstruation [22, 23]. Menstruation is a culturally sensitive topic and may not be freely spoken about in a group setting. IDIs were chosen to learn of individual experiences while managing menstruation and to draw out feelings and behavioural and cultural practices. The topic guides asked questions on girls’ personal experiences around menstruation and MHM and their views of how social and economic factors impact MHM. FGDs were used to explore girls’ shared view of the effect of the availability/non-availability of sanitary facilities and materials and the presence of boys on the management of their menstruation; and, their influence on school performance and attendance. Among boys, the FGD guide was used to learn about shared knowledge on menstruation and their attitude towards menstruating girls in the school setting. FGDs were most suitable for the purposes of understanding collective social views, values and norms [24, 25]. The KII guides for SHN coordinators, traditional leaders and female guardians focused on their knowledge, practices and attitude of adolescent girls towards MHM both at home and in schools. Acceptable and feasible strategies for healthy MHM in schools were also explored.

### Data analysis

The data from all the interviews and FGDs were analyzed using thematic analysis [26]. This comprehensive process involved reading through the transcripts for familiarization and then identifying key themes and codes that were entered into a codebook. Transcripts were entered into NVIVO, a qualitative software analysis package for coding themes with additional codes/themes emerging being added to the codebook [27, 28]. Indexing [29] was used to compare data within and between cases and between and within sources.

When analyzing to understand the issues surrounding MHM in the rural schools, four major themes emerged: Knowledge on menstruation and MHM, menstrual materials, School WASH facilities and school attendance as summarized in Table 2 below.

**Table 2:** Major and sub-themes that emerged from the data on the challenges girls face in practicing menstrual hygiene in rural schools

Major themes	Sub-themes
Knowledge on menstruation and MHM	• *Facts*
	• *Local understandings of menstruation*
Menstrual materials	• *Access*
	• *Absorbency*
	• *Availability in schools*
School water, sanitation and hygiene facilities	• *Water and soap*
	• *Privacy*
	• *Cleanliness*
School attendance and menstruation	• *Menstrual accidents at school*
	• *Girls’ behaviour during menstruation*
	• *Cultural views on school attendance during menstruation*

### Ethical considerations

The Ministry of General Education (MOGE) approved and commissioned the study. Ethical approval was obtained from the Excellence in Research Ethics and Science (ERES) Converge Institutional Review Board. The Ministry of Health (MOH) gave further approval to conduct the study with human subjects. Written informed consent were obtained from key informants and from guardians with assents obtained from pupils under 18 years old. Confidentiality of information was maintained by excluding any personal identifiers and the recorded data were stored in a safe place where no one except study staff had access.

## Results

The paper presents the results from the qualitative study using four main themes including *knowledge on menstruation and MHM, menstrual materials, school water, sanitation* and *hygiene facilities and school attendance and menstruation* along with quotes that support the findings.

### Knowledge on menstruation and MHM

#### Factual knowledge on menstruation

While the school curriculum covers menstruation as part of health science subjects, the majority of the girls did not know its physiological basis. Both teachers and girls reported lack of structured teaching on MHM. Girls explained that they learned about menstruation at menarche and did not understand why it happened with the majority reporting negative experiences of fear, worry, sadness and devastation at their first period:

*“Why we girls menstruate? I don’t know why.” (IDI, girl,15 years).*

“*I was devastated when I had my first period because I thought my blood was going to run out.” (IDI, girl, 14 years).*

Female guardians and traditional leaders revealed that grandmothers, mothers, aunties, older sisters and female teachers taught girls how to behave and take care of themselves during menstruation. Most female guardians and traditional leaders strongly forwarded the view that girls learn about menstruation and its paraphernalia only when they attain menarche with a view that it is when they will understand what is taught to them:

*“When they just reach puberty, that is when they are told about menstruation because before, she never saw blood. Now that she has seen the blood, that is when she can understand and learn.” (KII, guardian).*

#### Local understandings of menstruation

Traditional leaders, female guardians and girls narrated some of the teachings given to girls at menarche which revealed the local understanding of menstruation in the community. For instance, girls were told that when menstruating, they should not add salt while cooking as this causes chronic cough in those who eat the food. Additionally, some girls believed that being physically active during menstruation makes a girl menstruate for a longer period and that use of certain disposable sanitary pads cause cancer.

Girls preferred to dispose used menstrual materials in the pit latrine and not waste bins at school for fear that witches and Satanists would use the menstrual blood to bewitch them and rob them of their fertility:

*“You throw the used menstrual material in the toilet. If you throw it somewhere else apart from the toilet, some can use the same blood to bewitch you so that you experience stomach pains most of the time. You might not even have children of your own.” (FGD, girl).*

### Menstrual materials

#### Access to absorbent menstrual materials

Most girls reported that not having access to absorbent menstrual materials was a challenge to managing their menses. Girls commonly used cloth with only a few being able to access cotton wool from shops or cotton fields or able to afford disposable sanitary pads. The cloth they used was an old piece of used *chitenge* (thick sarong-like colourful multi-purpose fabric), blanket or general material which had to be washed for reuse or could be disposed of and replaced by another piece. Teachers reported that the cloth used may not be fit for purpose:

*“Sometimes you find that a child comes, messes up at school; and, when you try to find out what they use, it’s just a small piece of cloth whereby it can’t even hold the blood. So she just messes up and goes back home. So, I think the challenge is what to use.” (KII, female teacher).*

Girls reported that cloth filled up too quickly and did not stay in place by sticking to underwear leading to telling stains on their uniforms. Girls further reported that the cloth was malodorous when full, which some boys confirmed explaining that they were able to tell when a girl was menstruating from the smell:

*“If a girl is using pieces of cloth, and the piece of cloth has absorbed a lot of blood, the material starts to smell. So if you pass near her, the piece of cloth she is wearing will produce some bad smell.” (FGD, boys).*

On the other hand, some girls revealed that others may not have tight underwear or any underwear at all, making the use of menstrual materials and the menstrual experience very difficult.

Most girls reported that walking long distances to get to school when menstruating led to friction burns on their inner thighs from the wet and rough menstrual cloth. This discomfort discouraged girls from going to school when menstruating:

*“When you wear a piece of cloth and have to walk the long distance to school, you get burns between the thighs and this is worse when you keep the cloth for a long time. Sitting in class becomes difficult. Even before the 12:40pm knocking off time, you can find that you have messed up. So it is easy to change at home because you have cloth nearby.” (IDI, girl, 17 years).*

Despite most girls using cloth, they stated a preference for disposable sanitary pads especially when they are in school, due to their ability to absorb menstrual blood for longer periods, dry and comfortable feeling, and ease of disposal. Some girls had never seen a disposable sanitary pad despite having heard about it and the possible comfort it offers.

*“Pads suck blood and feel dry. They last up to 6 hours and you just feel comfortable. If you don’t have another one, you can wear one the whole day at school and you change later when you get home. But most of the girls cannot afford them because they are expensive and parents will tell you to use the cloth.” (FGD, girl).*

*“You cannot be free when you are using a cloth because it gets wet fast and you will sense that it’s now wet and sometimes it starts to smell. But if you put on a pad, you won’t feel it – you will be feeling the blood coming out but it won’t be wet. As a result, you will be more comfortable than using a cloth or wearing a lot of pants [underwear].” (FGD, girl).*

#### Availability of menstrual materials in school

Out of the six schools, only one stocked cotton wool in case of emergencies. The remaining schools provided toilet paper to absorb menstrual blood. Teachers reported that their schools do not have enough funds for menstrual pads.

*“The school usually buys cotton wool especially when there is a sport gathering and they give to the first aid department in readiness for an accident or if someone has ‘dripped.’ So they buy for everyone to use.” (FGD, girl).*

*“We only provide some tissues [toilet paper] for them, that’s what we can afford.”* (*KII, female teacher).*

*“You just have to carry material from home to change because you can’t find them at school.” (FGD, girl).*

### Water, sanitation and hygiene facilities in schools

#### Water and soap in the toilets

Direct observation showed that though all the schools had tap water, pit latrines did not have piped or stored water. Soap for cleaning hands or cleaning the facilities is generally not provided.

*“When a menstruating girl uses the toilet, she might leave some drops of blood in the toilet but you find that there is no water for her to clean it off or soap to wash her hands. So she would rather use the toilet at home.” (FGD, girl).*

While the two schools with SLTS programmes selected in the study had well-constructed toilets connected to the sewer system with enough space and privacy, they had no running water nor soap for hand washing. Due to this, most girls avoided use of the toilets, with some opting to wait until the end of the school day to use the toilet at home.

*“There are situations were cleaners don’t often clean the toilets and the toilets are spoilt and they smell…Even after using a toilet you can’t flush because water doesn’t get in” (IDI, girl, 14 years).*

*“Some girls throw pads in the flushable toilets and they get blocked and stop flushing” (FGD, girl).*

#### Privacy and toilet cleanliness

Girls found it difficult to maintain hygiene during menstruation at school because the toilets offered no privacy. Some schools had coed washrooms only. Observations showed that toilets were small with limited space. While they are designed to ensure privacy even without a door or lock with a wall screening the pit from the entrance into the toilet, girls did not feel safe from prying eyes when using these toilets for MHM purposes. Some girls said that they would rather not change the sanitary material until they got home. Teachers reported that most girls would rather stay home during menstruation so they can change comfortably while the girls who chose to attend school while menstruating would ask to use the teachers’ toilets which were clean and private.

*“The latrines at school have no doors; somebody can see you when you are inside so you can’t use them to change a menstrual pad.” (FGD, girl).*

*“The latrines have no doors; maybe we can love to have some doors … Especially [for] the girls, so that when one is inside they are able to lock.” (KII, female teacher).*

Some girls revealed that they do not use school toilets at all because they are too dirty mostly with faeces and urine all over the floor and walls and produced a very bad smell:

*“The toilets at school are very dirty. Some of the pupils here when they finish helping themselves in the toilet, they use the wall to clean themselves. And some girls when they are menstruating, they wipe blood on the walls. Others don’t even urinate or defecate in the hole so you find faeces and urine all over the floor and the toilets really have a bad smell, they stink!” (IDI, girl, 18 years).*

Observations of schools’ WASH facilities also showed that toilets in most of the schools did not meet World Health Organisation (WHO) functional standards because they had no doors, locks, anal cleansing materials, water, and soap or ash. The pupil to latrine ratio was very high in the selected study schools in the two districts being 110:1 in one district and 145:1 in the other.

### School attendance and menstruation

#### Menstrual accidents at school

Most girls reported that they stayed away from school when they were menstruating for fear of having a “menstrual accident” that stained their uniform. Pupils realized that the quality of their menstrual material was poor and most likely to stain their dresses. Both girls and teachers revealed that such stains caused a lot of embarrassment for girls and attracted teasing and laughter from other pupils, especially boys. Pupils highlighted one schoolgirl who was teased and never came back to school after that.

*“Girls are afraid to mess themselves up in class when they come with one pad and do not have the material to change. Others do not have the money to buy pads and only use pieces of cloth. So when they come to school and mess up on a desk, the boys begin to laugh.” (FGD, girl).*

*“Sometimes it happens that I am on my periods and I don’t have good materials to use, like pads. So I make sure I don’t go to school because I attend [bleed] for three days. And I don’t attend school for three days because if you don’t wear a pad, you will mess up and your friends will laugh at you! So, I go on the day after I am done.” (IDI, girl, 16 years).*

*“You can’t be free when you are menstruating like you always are when you are not [menstruating]. When you are menstruating and you are in class, maybe you do not sit properly and blood leaks on the pant [underwear] and out on the uniform. So you can’t be free, you will just be thinking that maybe you have messed up. So it’s better to be at home.” (FGD, girl).*

#### Girls’ behaviour at school during menstruation

Most of the girls revealed that they avoided interactions with others and participation in class activities when they were menstruating. The boys said that it was easy for them to tell when a girl was on her menses just by the way she restricted movements, outdoor play, talking and social interaction. Teachers reported that they had observed many girls appearing very uncomfortable and moody when they were menstruating to the point of not participating in class. One male teacher explained that girls leave his class without notice or disappear when they are on their periods and do not come back to school until the next day.

*“When a girl is on her period, she looks like a sick person. She doesn’t move up and down or jump around. She sits in one place in case she moves too much; she can drop the pad, that’s why she avoids this. She becomes quiet. She just remains seated in class.” (FGD, boy).*

*“Their behaviour changes when they are menstruating; you would notice that the girls become moody, they don’t want to talk. Sometimes they even become emotional especially to boys and won’t even participate in class. When you try to find out, she will tell you that she is not feeling well, that she has a stomach ache and as a teacher I would know that the girl is on her period.” (KII, male teacher).*

#### Cultural views on school attendance during menstruation

Traditional leaders and female guardians reported that they were aware of the many challenges girls faced during menstruation, such as absence of wash rooms at school that tested school attendance. Some parents encouraged girls to attend school and taught them how to take care of themselves and maintain personal hygiene to avoid smell and stains. Other parents instructed girls to stay at home until they finished menstruating to avoid any embarrassment resulting from menstrual leaks. Female guardians said that while girls were taught matters relating to marriage at menarche, emphasis was placed on the need for them to avoid ‘playing’ (a euphemism for sex) with boys as this would put them at risk of getting pregnant and drop out of school.

*“Sometimes when a girl is menstruating, the mother will tell her to stay at home to prevent them from messing up their uniforms at school and also avoid embarrassment. It is better to keep them at home until they finish menstruating because at school they have no place to go to wash the cloth and change.” (KII traditional leader).*

*“When girls become of age and start menstruating, we tell them that they are supposed to take care of themselves and we instruct them on how they are supposed to dress and wear materials so that people cannot know or tell that the girl is having her period. We also tell them that the moment you are going to have sex with a man you will become pregnant so concentrate on your education.” (KII, parent).*

## Discussion

This first qualitative study on MHM in rural Zambia found that girls could not maintain menstrual hygiene in school which restricted their school attendance and participation. Our findings show that girls did not know anything about menstruation before menarche and only received informal education on MHM when they attained menarche. Girls also skipped school due to fear of personal embarrassment and teasing from others, especially boys. As also shown elsewhere, inadequate MHM affected girls’ confidence, psychological wellbeing and ability to perform physical activities [9, 30]. School girls were unable to practice adequate MHM due to lack of preparedness for menarche, access to absorbent materials, and to water, soap and privacy while at school. Use of cloth also restricted their movements due to friction burns and insufficient underwear. It was clear that parents knew the difficulties their daughters faced during menstruation and, most supported and encouraged girls in every way possible to keep attending school.

Our study revealed limited knowledge and alternative views on the biological process of menstruation, usually learned after attaining menarche by the girls interviewed, findings similar to that reported for India by Thakre et al. [31]. This reveals the need to strengthen MHM in reproductive health education to empower girls through detailed MHM information in the school sexual reproductive and health education curriculum so that girls, boys and teachers are equally informed and can support girls to practice MHM effectively [5, 14, 32].

As found in Nigeria, Kenya and India, alternate views around MHM impacted girls’ choice of menstrual materials, where and how to dispose them, and whether to attend school or not during menstruation [30, 33, 34]. While some of the local views on menstruation could be meant to promote good MHM practices, such as the discrete disposal of used menstrual materials, there is still need to change some traditional practices to promote effective MHM among girls in schools.

With respect to menstrual material, schoolgirls rely heavily on cloth due to household poverty, which is similar to that found in Northeast Ethiopia where girls used pieces of cloth and rags because of lack of money [7]. In rural Malawi, cost of commercial pads was identified as one of the barriers to school attendance [9, 31]. Comfort during menstruation is essential if girls’ attendance is to be consistent for their improved educational attainment. This study has shown how cloth inhibits girls’ movements because of fear they will stain their dresses, a feeling stemming directly from the inadequate protection offered by menstrual cloth. However, while girls report difficulties in finding appropriate female hygiene products that can enable them to comfortably manage their menses and in turn encourage school attendance, provision of sanitary pads has produced mixed results in other settings [8, 35, 36]. This is an area worth further exploration in Zambia as some girls may not have appropriate underwear to hold sanitary pads. Given the need to keep menstruation private, the manner in which sanitary pads are provided may influence uptake. In our study context, provision of amenities would need to be discrete until education on menstruation makes it possible for girls to openly ask for and receive sanitary pads without embarrassment or fear of teasing.

Our findings also show that school WASH facilities are generally unable to support MHM. In the case of schools with SLTS, well-constructed toilet facilities with a connection to a sewer and water reticulation system have become “white elephants” as water provision is a challenge despite having enough space and privacy. This has also been found in other countries such as Nicaragua. Without water, these toilets become dirty quickly [37]. As reported by McMahon et al. from Kenya, poor WASH facilities deters girls from using the facilities at school, with most girls not using them at all and opting to stay home until they finish menstruating [38]. Efforts in school sanitation to address girls’ participation in education have largely ignored menstrual management in latrine design and construction with privacy and water availability remaining a challenge [39]. While it would appear intuitive, serious advocacy and open talk is needed between school authorities and relevant stakeholders to realize improvements in existing WASH structures to make them menstrual hygiene friendly and motivate menstruating girls to attend school regularly [9, 40, 41].

Our study has several limitations that should be noted. Our findings are limited to rural schools in Zambia; MHM issues in urban and peri-urban schools maybe different but equally important to understanding the extent of MHM challenges faced by schoolgirls. The study has also not provided an in-depth analysis on the differences between MHM practices in the schools with and without SLTS, in exception of the issues surrounding running water, space and privacy discussed for the two SLTS schools included in the study. Further, this study does not provide insights into school expenditure on MHM in relation to other school needs or quantify the effects of poor MHM facilities on school attendance. Also, we did not sample for information saturation nor did we assess it. However, the 12 IDIs with girls and the 25 KIIs yielded rich information, which is consistent with studies reporting on expected information saturation [42, 43]. However, this number may have been insufficient to reach meaningful saturation and identify meta-themes. We conducted 6 FGDs with girls and 2 with boys and the latter may have been insufficient to reach data saturation. Nonetheless, our study provides compelling evidence that the menstrual hygiene challenges experienced by schoolgirls in rural Zambia compromise their ability to participate in school activities and for some, their ability to attend classes. The robust qualitative design allowed the collection of lived experiences from girls and perspectives on MHM from teachers, boys, female guardians and traditional leaders. Physical inspection and assessment of the school WASH facilities allowed triangulation with girls, boys, and key informants narratives on MHM. This triangulation provides the evidence stakeholders need to ensure that girls have equitable access to amenities for optimal participation in education and self-development activities.

## Conclusion

Our findings suggest that girls in the rural schools are often forced to skip school due to the various challenges they face in practicing menstrual hygiene. Without absorbent and comfortable menstrual materials to use, girls are not able to perform to their full ability. To encourage girls to attend school, schools working with guardians, should provide absorbent sanitary pads and essential materials for reducing girls’ discomfort during menstruation. There is also need to improve the structure and maintenance of school WASH facilities to make them MHM friendly, including provision of adequate disposal systems and appropriate changing spaces within toilets. Teachers should work towards creating a supportive environment for girls that includes educating learners, including boys, to be understanding. Using the many valuable insights from this study, Zambian policy can be developed to make Zambia’s schools MHM friendly environments where girls can feel comfortable and safe whether menstruating or not.

## Additional files

Additional file 1: IDI guide for girls aged between 14 and 18 years. (DOCX 31 kb)

Additional file 2: FGD guide for girls aged between 14 and 18 years. (DOCX 38 kb)

Additional file 3: FGD guide for boys aged between 14 and 18 years. (DOCX 25 kb)

Additional file 4: KII guide for teachers. (DOCX 29 kb)

Additional file 5: KII guide for female parents/guardians. (DOCX 19 kb)

Additional file 6: KII guide for traditional leaders. (DOCX 28 kb)

## Abbreviations

DEDS: District Education Board Secretary
ERES: Excellence in Research Ethics and Science
FGD: Focus Group Discussion
IDI: In-depth Interview
KII: Key Informant Interview
MHM: Menstrual Hygiene Management
MOGE: Ministry of General Education
MOH: Ministry of Health
PTA: Parents’-Teachers’ Association
SHN: School Health and Nutrition
SLTS: School Let Total Sanitation
WASH: Water, Sanitation and Hygiene
