# Supplementary material for: Menstrual hygiene management in rural schools of Zambia: a descriptive study of knowledge, experiences and challenges faced by schoolgirls
Source: BMC Public Health. 2019 Jan 5;19:16. doi: 10.1186/s12889-018-6360-2 (PMC6321718; doi:10.1186/s12889-018-6360-2)
Supplement: Supplementary file 1 — IDI guide for girls aged between 14 and 18 years. (DOCX 31 kb) [file 12889_2018_6360_MOESM1_ESM.docx]

**IN DEPTH INTERVIEW GUIDE**

**Study Participants** - Female pupils aged between 14 and 18years that have commenced menstruation.

**Objectives:**

1. What evidence exists on various factors (social/demographic, economic, and cultural/traditional) that affect adolescent girls in relation to MHM?
2. Experiences and current knowledge and attitude of adolescent girls towards MHM both at home and in schools?
3. Determining how adolescent girls are affected by boy’s attitude towards menstruation
4. Generate evidence on the effects of menstruation on school attendance
5. To investigate the challenges girls have with managing their menses both at home and at school.

**Location:**

- Rufunsa District – Chipeketi and Chiyota Primary Schools and Rufunsa Secondary School.
- Mumbwa District – Mukanda and Keezwa Primary Schools and Nalusanga Secondary School.

**Date: _______________________________**

**Interviewer:__________________________**

**School Name:________________________**

**Time Start: _________________**

**Time End: __________________**

**SELF-INTRODUCTION**

My name is ______________ I am from CIDRZ along with my colleague here who will introduce herself. We are working together with the Ministry of Education. We would also like to know you, please introduce yourselves. Using a different name. You can pick a paper from tis box and the name written on the paper is what you will be referred to as.

**OPENING STATEMENT**

Welcome! We know as adolescents there are a number of things we go through including changes with our bodies. We would like to learn about these experiences so we can work together to support you as you go through these experiences. Your experiences and thoughts will go a long way in our efforts to support you. We will use a voice recorder to make sure we capture everything you say. Anything you say will be kept confidential your identity will not be revealed. The discussion will take a maximum of 1.5 hours.

| **Opening Questions - Personal Background and Questions about School**  *These questions are meant to build rapport.* | |
| --- | --- |
| QUESTIONS | PROBES |
| 1. How old are you? |  |
| 1. What grade are you in? | 1. How many pupils are in your class? 2. How many boys and girls? 3. Is your main teacher male/female? |
| 1. How long does it take you to travel to school? | - 1. How many minutes or hours?   2. Do you take a car, a bus or you walk?   3. Does anyone escort you to school? |
| 1. Who do you live with at home? | 1. How many brothers and sisters do you have? 2. Do you live with both parents? Aunties/Uncles? 3. Do you have a grandmother? How often do you see her? |
| 1. What do you like about school? | 1. What do you look forward to each time you’re going to school? 2. What is your favourite subject? 3. What don’t you like about school? 4. What would you change about school? |
| 1. How do you get along with your teachers? | 1. Are your teachers who you get along with male or female? 2. How does your teacher usually support you? 3. Are you free to talk to your teacher about your personal issues? |
| 1. What do you with your friends in your school during your free time? | 1. How many close friends do you have? Out of all your close friends how many of them do you share personal information with/trust? Why not the others? 2. In what ways do your friends help you? 3. Do you see them outside of school? Where? |

| **Knowledge on Menstruation**  *[These next few questions are going into more specific questions about menstruation. Are you ready to being?]]* | |
| --- | --- |
| Question | Probe |
| 1. When you hear the word menstruation, what goes through your mind? | - 1. What words do you and your friends use for menstruation? Any secret words?   2. What words do your female elders (grandmother/ mother/sisters/aunts) use for menstruation?   3. What terms do you use to refer to a girl that is currently menstruating? |
| 1. Can you tell me about the first time you **learned** about menstruation? | 1. How did it come up? 2. How old were you? How did you feel when you were told? [*Scared? Excited?*] 3. Who told you? 4. What did they tell you? 5. Did they tell you anything about how you should behave or act? 6. Did you learn about menstruation before or after your started your period? |
| 1. Why do women and girls menstruate? | *Information:*   1. When does it happen? How often?   *Symbolism:*   1. What changes in a girls’ life when she starts to menstruate? |

| **Personal Experience**  *These next few questions ask about your personal experience with menstruation. Let me know if you would like me to repeat or rephrase a question.* | |
| --- | --- |
| Question | Probes |
| 1. Tell me the story of the time you first got your period. | **Context**   1. How old were you? 2. What did you do? Did you know what to do? 3. Did you have the necessary materials? 4. Where did you get your period?   **Emotion**   1. How did you feel when you got your period?   **Practice**   1. What materials did you use? 2. Did anyone help you? Who? What did they do? 3. Where did you go to manage yourself? 4. Did you have everything you needed? If not, what did you need? What did you want?   **Social Support**   1. Who did you tell first? Why this person? 2. How did you tell this person? What did you tell them? 3. Did you try and keep it a secret? 4. Was there anyone you did not want to know? Why? 5. What did they tell you? How did they tell you to manage your period?   **Cultural Aspects**   1. Did you perform any traditional acts when you started menstruation? What meanings do the acts have? 2. Who performed them? Who was with you? 3. What lesson are they teaching? |
| 1. Now think about your most recent period, how did you manage it? | **Context**   - 1. Where were you when you first realized you got your period?   2. What did you do?   3. Were you prepared? Did you have supplies with you? What supplies?   4. Where did you get those supplies? Where do you keep them?   **Practice**   - 1. Are you able to tell when your period is coming? How? Does this help your prepare?   2. What materials did you use? Why these materials? Do you normally use these materials?   3. Do you use different materials on different days? Why?      1. At different times? Why?      2. At different places? Why?   4. Any materials you do not use?   5. How often do you change your material on your heaviest day?   **IF DISPOSAPLE MATERIALS (pads, wool cloth, etc.):**   1. How do you get these materials? 2. Does someone purchase them for you? Who? Where? 3. How much are they? 4. How do you dispose of them? 5. Is there any way you should not dispose of these materials? Why? 6. Do you every buy reusable pads? When? Why or why not?   **IF REUSABLE MATERIALS:**   1. How do you get these materials? 2. Does someone purchase them for you? Who? Where? 3. How much are they? 4. How do you maintain these materials? How is it washed? With soap?    - Dried? Where do you dry these materials? 5. Do you ever buy disposable pads? When? Why or why not?   **IF OTHER MATERIAL:**   1. How do you get these materials? 2. Does someone purchase them for you? Who? Where? 3. How much are they? 4. How do you maintain these materials?    1. Where do you clean yourself when you are menstruating? Do others also use this place? Who?    2. Do you have access to:       1. Water (hot/cold)?       2. Soap       3. Privacy when managing your period?    3. Do you experience abdominal pain? How severe are they?    4. What would make managing your period at home easier? Any challenges? |
| 1. How do you manage your period at school? | **Emotions**   1. How do you feel going to school when you are menstruating? [*Comfortable? Nervous?]*   **Practice:**   1. What materials do you use at school? Are these materials different from what you use at home? 2. At school, where do you go to change your material? 3. Is there water, soap, privacy?   **IF DISPOSAPLE MATERIALS (pads, wool cloth, etc.):**   1. Where do you get these materials? 2. How do you dispose of these materials at school? 3. Is there any way you should not dispose of these materials at school? Why?   **IF REUSABLE MATERIALS:**   1. Where do you get these materials at school? 2. How do you maintain these materials at school? 3. Do you wash at school? Where? How is it washed? Wash with soap?    - Dried? Where do you dry these materials at school? 4. How do you carry used reusable materials?   **IF OTHER MATERIAL:**   1. How do you get these materials at school? 2. How do you maintain these materials? 3. Do you have access to sanitary materials at schools?    - Where do you get them?    - What materials are available?    - Do you have to ask someone? Who?    - How often can you get them?    - How many can you get?    - Is there a certain time you can access these materials? When? 4. How does the distance you travel from home influence how you manage your period at school? 5. How do you manage cramp/ dysmenorrhea during school? 6. What do you find challenging about managing your menses at school? |
| 1. Can you tell me about a time when you had your period at school and you weren’t prepared or did not have supplies?   OR  If they do not have a story about this experience, ask what they would do? | **Context:**   - 1. What happened?   2. How old were you?   3. What happened?   **Emotions**   - 1. How did you feel?   **Practice**   - 1. What did you do first?   2. Did you tell anyone? Who?   3. Where did you go? Home? Stay at school?   4. What material did you use?   5. Where did you get it from? Who gave it to you?   6. Where did you go to clean to yourself?   7. Was there water/soap/privacy?   **Social Support**   1. What advice have you been given on messing up your uniform while at school? 2. Did anyone help you that day? Who? What did they do?   **School Challenges:**   1. Did you stay in school that day? 2. Was anything difficult for you at school? 3. Who did you not want to know you were menstruating? Why? |

| **Closing Questions**  *Great, now we’re almost done. Before we finish, I want to get recommendations from you on how to improve your ability to manage your period at school. We will share your recommendations with the Ministry of Education* |
| --- |
| 1. How can your school best support you manage your menstruation? |
| 1. How do you think your teachers can best support you to manage your menstruation? |
| 1. What can parents/families do to support girls? |
