# Supplementary material for: Menstrual hygiene management in rural schools of Zambia: a descriptive study of knowledge, experiences and challenges faced by schoolgirls
Source: BMC Public Health. 2019 Jan 5;19:16. doi: 10.1186/s12889-018-6360-2 (PMC6321718; doi:10.1186/s12889-018-6360-2)
Supplement: Supplementary file 2 — FGD guide for girls aged between 14 and 18 years. (DOCX 38 kb) [file 12889_2018_6360_MOESM2_ESM.docx]

**FOCUS GROUP DISCUSSION GUIDE**

**Additional Probes**

**Study Participants** - Female pupils aged between 14 and 18 years that have commenced menstruation.

**Sample:** 48 (8pupils x 6schools)

**Objectives:**

1. To explore whether MHM practices are related to adolescent girls’ school attendance.
2. To understand how adolescent girls are affected by the availability of water supply and sanitary facilities and materials (such as toilets, hand washing devices, sanitary pads, materials and soap) during menstruation.
3. To determine acceptable and feasible strategies promoting healthy MHM practices can be implemented in schools.

**Location:**

- Rufunsa District – Chimusanya, Chiyota and Rufunsa Primary Schools.
- Mumbwa District – Matala Primary School, Kasalu basic school and Kalilwe Secondary School.

**Date: _______________________________**

**Moderator:___________________________ Note Taker:___________________________**

**School Name:________________________**

**Time Start: _________________**

**Time End: __________________**

**SELF-INTRODUCTION**

My name is ______________ I am from CIDRZ along with my colleague here who will introduce herself. We are working together with the Ministry of Education. We would also like to know you, please introduce yourselves. Using a different name. You can pick a paper from tis box and the name written on the paper is what you will be referred to as.

**OPENING STATEMENT**

Welcome to this group discussion. We know as adolescents there are a number of things we go through including changes with our bodies. We would like to learn about these experiences so we can work together to support you as you go through these experiences. Your experiences and thoughts will go a long way in our efforts to support you. We will use a voice recorder to make sure we capture everything you say. Anything you say will be kept confidential your identity will not be revealed. The discussion will take a maximum of 1.5 hours.

**SECTION A – ICE BREAKER**

- Start by singing a famous song known to all the pupils.
- Tell us what you would like to work as when you grow up and give a reason for your answer

| **Opening Questions**  *In these first few questions, we will talk about school and what you learn at school.* | |
| --- | --- |
| **Questions** | **Probes** |
| 1. What do you like about school?   *[Friends, teachers, subjects*] | 1. What are some reasons students do not go to school sometimes?    - Anyone who discourages you from attending school? Why? Who? 2. Who encourages pupils to always attend school? |
| 1. What are girls first taught about menstruation? | 1. At what age are girls usually taught about menstruation? *[Probe: before or after they start menstruation]* 2. Who is responsible for teaching young girls about menstruation? 3. What health education do students receive here at school?    - What grade is it taught?    - Who is responsible for teaching this topic?    - What topics are discussed?  - Are boys and girls taught together? |
| 1. What is challenging about menstruating? | 1. What cultural practices do girls observe when on their menstruation? 2. How are girls expected to behave once they have started menstruating? How is their behaviour different from before? 3. How do girls manage menstrual pain: when at school? At home? 4. Do girls miss school because of menstruation? Why or why not? |

| **KEY ACTIVITY – Scenario: Menstruation During a School Term** | |
| --- | --- |
| ***Aim:*** *this exercise is designed to understand what young girls go through while menstruating on a school day. The facilitator will read out a scenario that will be supported by a story board to help the girls understand.*  ***Scenario****: We are going to start talking about a gril’s experience of menstruation. Imagine a girl in your school name Jelita. Jelita is 15 years old. This morning, she wakes up in the morning and she realizes that she has started her periods. She goes to bath and gets ready for school. She goes to school and sits in class. Jelita asks her teacher to go the toilet at around 10:00am, she goes to check herself and change. In the afternoon, Jelita is playing outside. Her friend Mainza then sees a red spot on her dress.*  **What’s Needed**: Story board depicting the scenario. | |
| **Scenes** | **Questions and Probes** |
| **Scene 1:** Jelita wakes up | 1. Please describe what you see in this picture.    1. What is happening? |
|  | 1. How does Jelita feel when she realizes she has started her period when she wakes up? [*Happy, excited, confusion*]    1. What do you think is the first thing she will do when she realizes she has started her period?    2. Do you think she will tell anyone? If yes, who?       - - Will her father know that the she is on her period?         - What about her brother?    3. Now that Jelita is on her period, how does she feel about going to school?    4. If Jelita’s mother or guardian know that she is on her period, will they allow Jelita to go to school? Why or why not? |
| **Scene 2:** Jelita prepares herself for school | 1. Think about the way Jelita gets ready for school everyday. Does she get ready ant different now that she is on her period?    1. Is it important that she bathe?    2. Where does she take her bath from while she is on her period?    3. Do other people also take baths in the same place where she takes a bath? Why or why not? |
|  | 1. What material does Jelita use?    1. Why is she using this material? Who do you think taught her to use this material?    2. How comfortable is the material Jelita is using? Would Jelita prefer to use another material? If so, what material and why? |
|  | 1. **Scene 3:** Jelita is at school Once Jelita is in the classroom:    1. How is her concentration? Participation?    2. b. Who at school do you think she told that she is on her period? Boys?    3. How is her behaviour towards her male friends? Her Her teacher? |
| **Scene 4:** Jelita goes to use the latrine at school | 1. Please describe what you see in this picture.    1. What is happening? |
|  | 1. If Jelita wants to go and check on herself:    1. Are there any rules around leaving the classroom during class? What rules?    2. Is her teacher (probe: male and female) supposed to know why she is going to the toilet? Why?    3. Do you think her teacher is ok with her leaving the room?    4. How possible is it for Jelita to change her material at school? What are the challenges? |
| **Scenario 5:** Jelita is playing outside and has a red spot on her dress by her bum. Her female friend noticed the spot and has told her. | 8. Please describe what you see in this picture.   1. How does Jelita feel when her friend tells her that she has a spot on her back? (Scared, embarrassed, shy, afraid) 2. What is the first thing Jelita will do when she finds out she has a red spot on her back? 3. What will she do to manage the spot on her dress? *[Is she thinking of leaving school?]*    1. Where will she go?    2. Who will she talk to? |
|  | 1. If boys see the red spot on her dress,    1. How do you think the boys will react? Why will they react this way?    2. If other girls see the red spot on her dress, how do you think they will react? 2. What will her mothers/grandmother do if they know that Jelita has messed up her uniform? |

| **Understanding Facilities at School**  *Great, let’s continue. These next couple questions ask about the facilities available at this school.* | |
| --- | --- |
| **Questions** | **Probes** |
| 1. Please describe the usual condition of the latrines/facilities at this school?  Cleanliness? Smell? Privacy (locked/unlocked)? Safety? | 1. How many latrines are there? How many of them can you use? 2. Are there separate latrines for boys and girls? 3. What materials are available for personal hygiene? [*Soap/water/materials/tissue/handwashing stations*] 4. Where are the latrines located from your class? 5. Does everyone use the latrine? Where else do others go? 6. How comfortable do you feel using these latrines? |
| 2. How do girls utilize the toilet when they are menstruating? | 1. Are you able to clean yourselves with the current toilet structure? 2. Do girls feel safe when they use the latrine? Why or why not? 3. Do girls visit the latrine with a friend? 4. What things do you feel should be in the toilet to help manage menstruation? |

| **KEY ACTIVITY – MENSTRUAL MANAGEMENT MATERIAL ACTIVITY** | |
| --- | --- |
| **Activity Summary:**  This activity involves showing girls a variety of materials for managing menstruation. The products selected should be include items that are typically used (like cloth or cotton wool) as well as products that may be hard to get (disposable sanitary napkins) or that may not be in use but are of interest (lie reusable sewn sanitary napkins). They should be able to touch them and look at them closely. Girls will then be asked a series of questions about the items they are shown.  **Activity Goal:**  The goal of this activity is to understand what girls think about a variety of materials so that any recommendations for materials are well informed.  **Activity Strengths:**  By presenting many materials together, you can have the girls compare and contrast the items. It also eliminates bias as not just one material (like commercial pads) is being queried. | |
| **Key Questions** | **Probes/Follow-ups** |
| 1. Now I’d like to show you a few materials.  *Show the girls several materials: commercial sanitary napkin, reusable sanitary napkin, cloth, toilet paper, cotton, pants, whatever else girls in your community may use, etc.*  *Ask the probing question regarding each material first and then move on to the next material. .* | **FOR ALL MATERIALS:**   1. Can someone explain to me what this is? How is it used? 2. Is this material available in your community? 3. How effective is this material for managing menstruation? 4. What do you think of using this material in school? 5. What do you think of changing this material in school? 6. How do girls know when to change this material? How often are they supposed to change do you change this material? 7. How expensive are these materials? 8. Where do they purchase the material? 9. Who purchases them?   **FOR REUSABLE MATERIAL (CHITENGE):**   1. When is this material supposed to be cleaned? How is this material cleaned? With water? With soap? 2. How is this material dried? Where is it dried? 3. Where is the material stored? 4. Is the material ever thrown away?   **FOR DISPOSABLE PADS/TAMPONS/COTTON WOOL:**   1. Where are these pads normally disposed? Why? 2. How expensive is the material? |
| 2. How many different materials do girl use at certain times of your period? | 1. What affects girls’ choice of material? 2. Do girls use different materials at home? 3. Do you use different materials at different times during your menses? Why? |
| 3**.** How do girls access these materials at school? | 1. What materials are available at school? 2. Where at school do you find the material? 3. How many materials can a girl get at one time? 4. Can you get these materials at any time? What time? |

| **Closing Questions**  *Great, now we’re almost done. Before we finish, I want to get recommendations from you on how to improve your ability to manage your period at school. We will share your recommendations with the Ministry of Education.* | |
| --- | --- |
| **Questions** | **Probes** |
| 1. What information would you like the school to provide on menstruation? | 1. Is there any information you would like to be taught on menstruation in your school?    - Who, at school, is the best to teach this? 2. Should boys learn about menstruation?    - What would you like them know? 3. When should boys learn about menstruation? Who should teach them? |
| Do you have any questions? | |
| *Thank you so much for your time! You have provided us with real valuable information. This information is useful for the ministry when they make decisions.* | |
