# Supplementary material for: Menstrual hygiene management in rural schools of Zambia: a descriptive study of knowledge, experiences and challenges faced by schoolgirls
Source: BMC Public Health. 2019 Jan 5;19:16. doi: 10.1186/s12889-018-6360-2 (PMC6321718; doi:10.1186/s12889-018-6360-2)
Supplement: Supplementary file 3 — FGD guide for boys aged between 14 and 18 years. (DOCX 25 kb) [file 12889_2018_6360_MOESM3_ESM.docx]

**FOCUS GROUP DISCUSSION GUIDE**

**Additional Probes**

**Study Participants** – Male pupils aged between 14 and 18years

**Sample:** 16 (2 FGDs x 8people)

**Objectives:**

1. Determining the attitude of boys in schools towards menstruation

**Location:**

- Rufunsa District – Chimusanya, Chiyota and Rufunsa Primary Schools
- Mumbwa District – Matala primary, Kasalu basic Primary Schools and Kalilwe Secondary School.

**Date: _______________________________**

**Moderator:___________________________ Note Taker:___________________________**

**School Name:________________________**

**Time Start: _________________**

**Time End: __________________**

**SELF-INTRODUCTION**

My name is ______________ I am from CIDRZ along with my colleague here who will introduce herself. We are working together with the Ministry of Education. We would also like to know you, please introduce yourselves. During this discussion you will use numbers for identification.

**OPENING STATEMENT**

Welcome to this group discussion. We know as adolescents there are a number of things we go through including changes with our bodies. We would like to learn about what you know about puberty for both boys and girls. We will use a voice recorder to make sure we capture everything you say. Anything you say will be kept confidential your identity will not be revealed. The discussion will take a maximum m of 1 hour. Do you have any questions before we begin?

**SECTION A – ICE BREAKER**

- Start by singing a famous song known to all the pupils.
- Tell us what you would like to work as when you grow up and give a reason for your answer.

| **Knowledge and Behavior**  *We will begin by asking questions about becoming of age.* | |
| --- | --- |
| Question | Probe |
| 1. What do you understand by the term puberty (becoming of age)?   Puberty = transition from childhood into adulthood | 1. Does it mean the same for boys and girls?    - What’s the difference? 2. What words do you use when you are talking about puberty for boys? What words for girls? 3. What do they mean? 4. Where did you learn these words? |
| 1. Have you heard about the term menstruation? What do you understand by it?   *If there are some in the group that don’t know what menstruation is, explain it briefly* | 1. Where did you learn about menstruation?    - At school? Which subject?    - At home? Who taught you? 2. Is it acceptable for boys to talk about menstruation? 3. Who talks to boys about menstruation?    - When did boys first learn about menstruation?    - Do parents talk about menstruation with their sons?    - Do girls talk to boys about menstruation? If so, what do they say? 4. How often does it occur in a girl’s life? 5. What do you think girls experience during menstruation? 6. What products do girls use when they are menstruating? |
| 1. How do girls behave when they are menstruating?    - At break time?    - In the class room? | 1. Is this different from their normal behavior (when girls are not menstruating)? How so? 2. How do you know that a girl is menstruating? 3. Why do you think she behaves differently? |
| 1. Can you tell me how boys behave towards girls when they are menstruating? | 1. Did anyone teach you how to behave? Who? 2. Why would they behave in that particular way? 3. What if the girl is a relative, how would they behave? 4. Why do they behave like this when you know a girl is menstruating? Does anyone tell them how to behave? 5. Have you ever come across a situation where a girl was teased when she was menstruating? What happened?    - Who was teasing the girl?    - What did the girl do? Did she leave school? When did she come back?    - Did the teacher do anything? 6. When this happens at happens at school, does this effect other girls? How? 7. Would you feel comfortable supporting your female friends at school? How? What could you do? |
| 1. Would boys like to know more about menstruation? | 1. What in particular would they like to know?    - How should they learn? In classes with girls? Just boys? With female teacher? Male teacher? |
|  | |
| **Closing**  *Great, now I want to get some of your recommendations* | |
| 1. How can teachers help pupils on issues of menstruation? | |
| 1. If the school had a programme targeted to helping girls while on their menstruation, how could boys be involved? How do you feel about boys being involved in the production of girl sanitary wear? | |
| 1. Do you have any question? | |
| *Thank you all for your time.* | |
