# Supplementary material for: Menstrual hygiene management in rural schools of Zambia: a descriptive study of knowledge, experiences and challenges faced by schoolgirls
Source: BMC Public Health. 2019 Jan 5;19:16. doi: 10.1186/s12889-018-6360-2 (PMC6321718; doi:10.1186/s12889-018-6360-2)
Supplement: Supplementary file 4 — KII guide for teachers. (DOCX 29 kb) [file 12889_2018_6360_MOESM4_ESM.docx]

**KEY INFORMANT INTERVIEW GUIDE**

**Additional Probes**

**Date: _______________________________**

**Study Participants** –Female Parents

**Objectives:**

1. To determine acceptable and feasible strategies promoting healthy MHM practices can be implemented in schools.
2. To determine the experiences and current knowledge and attitude of adolescent girls towards MHM both at home and in schools

**Location:**

- Rufunsa District – Chimusanya, Chiyota and Rufunsa Primary Schools.
- Mumbwa District – Matala primary School, Kasalu basic school and Kalilwe Secondary School.

**Interviewer:___________________________**

**School Name:________________________**

**Time Start: _________________**

**Time End: __________________**

**SELF-INTRODUCTION**

My name is ______________ I am from CIDRZ along with my colleague here who will introduce herself. We are working together with the Ministry of Education. We would also like to know you, please introduce yourselves. Using a different name. You can pick a paper from this box and the name written on the paper is what you will be referred to as.

**OPENING STATEMENT**

Welcome! We know that young girls go through a number of changes in their lives when they are growing into adults. We would like to learn about these experiences from you as a parent/guardian so we can work together to support young girls in schools as they go through these experiences. Your experiences and thoughts will go a long way in our efforts to support young girls. We will use a voice recorder to make sure we capture everything you say. Anything you say will be kept confidential your identity will not be revealed. The discussion will take a maximum of 1 hour.

| Questions | Probes |
| --- | --- |
| 1. How do you know /tell that a girl has reached puberty? | 1. In what ways do you prepare girls for puberty? |
| 1. How did your daughter first learn about menstruation? | 1. Who taught her? Anyone else? 2. If it was you, what exactly do you teach them about? What information did you share with her when she started?    - How comfortable were you talking to you daughter menstruation?    - How comfortable are other mothers? 3. In general, when are girls talked to about menstruation? Why at this time? |
| 1. Can you tell me what happened when your daughter first got her period? | 1. How did you find out? 2. What did you do? 3. Who did you share this information with? [*Husband, female relatives, school teachers, siblings?]* 4. What cultural or traditional practices are performed when your daughter first got her period? How do you feel about these traditions? |
| 1. Are children provided puberty education at home? | For Girls:   1. What is taught? Who teaches them? [*Mothers? Fathers? Aunts? Grandmothers?]* 2. When are they taught?   For Boys:   1. What is taught? Who talks to boys? [*Mothers? Fathers? Uncles?*] 2. When are boys taught? 3. Do parents/guardians talk to them about menstruation? Why? Why not? 4. Any other information provided? |
| 1. In your community, how are girls taught to manage their menstruation?    - Personal hygiene    - Disposal/drying | 1. How do girls manage their personal hygiene? 2. What materials are girls taught to use? 3. What materials do girls actually use? Are different materials used on different days or times? 4. If disposable:    - How is this material disposed? *[Burning, burying]*    - Who provides the funds? Why? 5. If reusable:    - How is the material washed?    - How is the material dried? Where?    - Who provides the funds? Why? 6. If other:    - How is this material washed or disposed? 7. What’s your preferred material for your daughter to use? Why? Does she use this material? If not, why? 8. How is a girl taught to manage their hygiene during menstruation?    - Can a girl do without water and soap while they are on their menstruation? How? 9. What challenges do they face managing their menstruation while at home? 10. At school? |
| 1. How should daughters behave during their menstruation? | 1. How does her behaviour change from before she started her period and after she started her period?    - At home? 2. Why does her behavior change from before and after she starts her period?    - At home?    - At school? 3. How should they behave towards their fathers? Any reason for this? 4. How should they behave with    - Male friends?    - Brothers?    - Family?    - Female friends? 5. Any traditional or religious restrictions? What are these? |
| 1. How do you support daughter/s through menarche and monthly periods? | 1. What should be in place at home to support your daughters during menstruation? 2. How does the father support the daughter through menarche and menstruation?    - Who tells the father that the daughter has began menarche?    - What is the father expected to do at the time that he knows the daughter has began menarche?    - What’s the difference in the support he provides and the support you provide? Financial support?    - Does the father have expectations of the daughter once they begin menstruating? |
| 1. Are there times when your daughter misses school during her menses? | 1. Why does your daughter miss school during her menses? 2. Are they allowed to attend school while on their period? Why not? 3. If your daughter messes up herself while at school, how can this make you feel? What would you do to her? 4. How often do you think your daughter misses class during menstruation? 5. What things should a school have in place to help a girl manage her menstruation?    1. Sanitation facilities    2. Menstrual materials    3. Education |
| What recommendations do you have on how mothers can best support their girls during menstruation? How about fathers? And elders?  What recommendations do you have on how schools can best support girls during menstruation?  Do you have any questions for me? | |
| Thank you for your time | |
