# Supplementary material for: Menstrual hygiene management in rural schools of Zambia: a descriptive study of knowledge, experiences and challenges faced by schoolgirls
Source: BMC Public Health. 2019 Jan 5;19:16. doi: 10.1186/s12889-018-6360-2 (PMC6321718; doi:10.1186/s12889-018-6360-2)
Supplement: Supplementary file 5 — KII guide for female parents/guardians. (DOCX 19 kb) [file 12889_2018_6360_MOESM5_ESM.docx]

**KEY INFORMANT INTERVIEW GUIDE**

**Study Participants** – Traditional Leaders

**Objectives:**

1. To determine acceptable and feasible strategies promoting healthy MHM practices can be implemented in schools.
2. To determine the experiences and current knowledge and attitude of adolescent girls towards MHM both at home and in schools

**Location:**

- Rufunsa District – Chipeketi and Chiyota Primary Schools and Rufunsa Secondary School.
- Mumbwa District – Mukanda and Keezwa Primary Schools and Nalusanga Secondary School.

| # | Questions | Probes |
| --- | --- | --- |
| 1 | Culturally in your context, what does it mean when a child becomes of age? | - What does it mean for boys? For girls? How are they different? - What traditions are associate with coming of age here? - Is the whole community involved in the coming of age process? |
| 2 | Specifically looking at girls, what is expected of them when they become of age? | - What cultural beliefs does your community uphold about girls and menstruation? - How are girls expected to behave once they have started menstruation? - How are girls in this community treated once they have started menstruation? Do people know when a girl has started? How? |
| 4 | How do you think girls in your community manage their menses at school? At home? | - Do you believe they have access to the necessary materials? (facilities, materials, education) - What challenges do you think girls face in managing their menses especially when in school? - Are girls prepared to manage their menses in this community at home and at school? |
| 6 | Do cultural/ traditional beliefs have any barriers on MHM and girl attendance in schools? How? | - How do you address these issues at the school levels? |
| 8 | What programs or initiatives are there at community level supporting girl attendance in school? |  |
| 10 | What do you suggest should be done to improve MHM in schools? |  |
| Do you have any questions for us? | | |
| Thank you | | |
