# Supplementary material for: Menstrual hygiene management in rural schools of Zambia: a descriptive study of knowledge, experiences and challenges faced by schoolgirls
Source: BMC Public Health. 2019 Jan 5;19:16. doi: 10.1186/s12889-018-6360-2 (PMC6321718; doi:10.1186/s12889-018-6360-2)
Supplement: Supplementary file 6 — KII guide for traditional leaders. (DOCX 28 kb) [file 12889_2018_6360_MOESM6_ESM.docx]

**KEY INFORMANT INTERVIEW GUIDE**

**Study Participants** – Teachers

**Objectives:**

1. To determine acceptable and feasible strategies promoting healthy MHM practices can be implemented in schools.
2. To determine the experiences and current knowledge and attitude of adolescent girls towards MHM both at home and in schools

**Location:**

- Rufunsa District – Chipeketi and Chiyota Primary Schools and Rufunsa Secondary School.
- Mumbwa District – Mukanda and Keezwa Primary Schools and Nalusanga Secondary School.

**Instructions:**

- Talk to the head teacher first and ask to interview the School Health and Nutrition Coordinator (SHN). If SHN coordinator is not available or the position does not exist at school, ask for a teacher who manages the school health programs.

**Date: _______________________________**

**Moderator:___________________________ Note Taker:___________________________**

**School Name:________________________**

**Time Start: _________________**

**Time End: __________________**

| **Health Education Programs at School**  *These first few questions are about health education programs offered at school.* | |
| --- | --- |
| **Questions** | **Probes** |
| 1. What health programs are available at this school? For example, some schools have school health and nutrition (SHN) programs? | 1. What topics are covered? 2. Is reproductive health/sexual education part of the school curriculum? 3. When is this taught? What age, class or age group? Before or after school? 4. Who is responsible for providing this education? 5. What is taught? What topics are discussed? 6. What materials are available to pupils on reproductive health? 7. How do you work with other health facilities in your area? What specific areas do you collaborate? |
| 1. What menstrual hygiene management education do pupil receive? | 1. When is this taught? What age, class or age group? Before or after school? 2. Who is responsible for providing this education? 3. What is taught? What topics are discussed? 4. Are boys and girls taught together during menstruate education? 5. How comfortable are teachers in providing menstrual hygiene education to female pupils? Do male and female teachers feel differently? 6. How do you feel about menstrual specific information should be discussed in schools? How do you feel other teachers feel about this?   IF NO MENSTRUAL HYGIENE EDUCATION:   1. If menstruation education is not provided, do girls receive any educational materials? Such as booklets?   IF MALE TEACHER: How do you personally feel about teaching menstrual hygiene education to female students? |

|  | |
| --- | --- |
|  |  |
| 1. How do girls manage their menstruation while at school? | 1. What specific difficulties do you believe they face? WASH facilities, privacy, education, materials? 2. How do you think the girls manage these difficulties? 3. What facilities [*sanitation, water, handwashing*] are available for girls to use while on their menstruation? 4. Do girls and boys have separate facilities? How are these facilities different? Do girls use the provided facilities? 5. Do female teachers have access to these facilities as well? Do they have separate facilities? 6. How appropriate are the current facilities for girls to use during menstruation? For teachers?    - Why are they appropriate? Why not?    - What could be changed to make them more appropriate? |
| 1. Can you tell me how female pupils behavior changes when they are menstruating? | 1. Do you recognize when a female pupil is menstruating in class? If so, how? 2. Does this change in behavior influence girls’ attendance rates? 3. How do you compare attendance rates between boys and girls? 4. What are the most common reasons girls usually do not attend classes in comparison to boys? 5. What role do you think menstruation plays in girls missing class? 6. What can teachers do to ensure that girls attend classes even though they are menstruating?    - - What can the school do?      - What can parents do? 7. In some schools, girls are bullied and teased if people know they are menstruating. Are you aware of such incidents? What can you tell me about bullying and teasing that may happen at this school? |
| 1. Does the school provide sanitary materials for those girls who may start menstruating while at school? Please explain how a girl can access these materials at the school. | 1. Where does she get them? 2. What materials are available? 3. Who provides these materials to girls? Who do girls have to talk to? 4. How many materials are provided? How often can a girl get them? 5. Is there only a certain time girls can access these materials? When? |
| 1. What difficulties do you face when managing girls who are menstruating in class? | 1. Do you feel prepared to manage these situations? Why or why not? 2. Who at the school handles menstrual related cases? For example, if girl has messed up her uniform, who does she see at the school? 3. How are these cases handled? 4. What support does this person offer to girls?   IF NO FOCAL PERSON   1. If there is no focal person, which member of staff in the school do you feel can be placed as a point person to handle menses issues? Why this person? 2. How can they assist girls with menstrual related challenges? |
| **FOR MALE TEACHERS ONLY:**   1. How do you handle girls who are menstruating in your class? | 1. Do you do anything differently than if she was not menstruating? 2. In class, have you ever had to handle a case where girls soil their uniforms with menstrual blood? What did you do? |

| **Closing**  *Thank you so much for your time with this! Before we wrap up, I want to get your recommendations to share with the Ministry of Education.* |
| --- |
| 1. Are teachers appropriately trained to manage menstruation in schools? What training should be provided? |
| 1. Male teachers and male nurses are important part of the school community. How can they best be involved? Are male teachers facing any challenges when supporting girls during their menstruation? How can they be supported? |
| 1. How can parents be involved in school programming to help support girls? |
| 1. Do you have any questions for me? |
